# Computational prediction of promotors in *Agrobacterium tumefaciens* strain C58 by using the machine learning technique

**DOI:** 10.3389/fmicb.2023.1170785

**Published:** 2023-04-13

**Authors:** Hasan Zulfiqar, Zahoor Ahmed, Bakanina Kissanga Grace-Mercure, Farwa Hassan, Zhao-Yue Zhang, Fen Liu

**Affiliations:** ^1^Yangtze Delta Region Institute (Huzhou), University of Electronic Science and Technology of China, Huzhou, China; ^2^School of Life Science and Technology and Center for Informational Biology, University of Electronic Science and Technology of China, Chengdu, China; ^3^Department of Radiation Oncology, Peking University Cancer Hospital (Inner Mongolia Campus), Affiliated Cancer Hospital of Inner Mongolia Medical University, Inner Mongolia Cancer Hospital, Hohhot, China

**Keywords:** prokaryotic promotors, feature extraction, *agrobacterium tumefaciens* strain C58, feature selection, algorithms

## Abstract

Promotors are those genomic regions on the upstream of genes, which are bound by RNA polymerase for starting gene transcription. Because it is the most critical element of gene expression, the recognition of promoters is crucial to understand the regulation of gene expression. This study aimed to develop a machine learning-based model to predict promotors in *Agrobacterium tumefaciens* (*A. tumefaciens*) strain C58. In the model, promotor sequences were encoded by three different kinds of feature descriptors, namely, accumulated nucleotide frequency, *k*-mer nucleotide composition, and binary encodings. The obtained features were optimized by using correlation and the mRMR-based algorithm. These optimized features were inputted into a random forest (RF) classifier to discriminate promotor sequences from non-promotor sequences in *A. tumefaciens* strain C58. The examination of 10-fold cross-validation showed that the proposed model could yield an overall accuracy of 0.837. This model will provide help for the study of promoters in *A. tumefaciens* C58 strain.

## 1. Introduction

**Figure 1:**
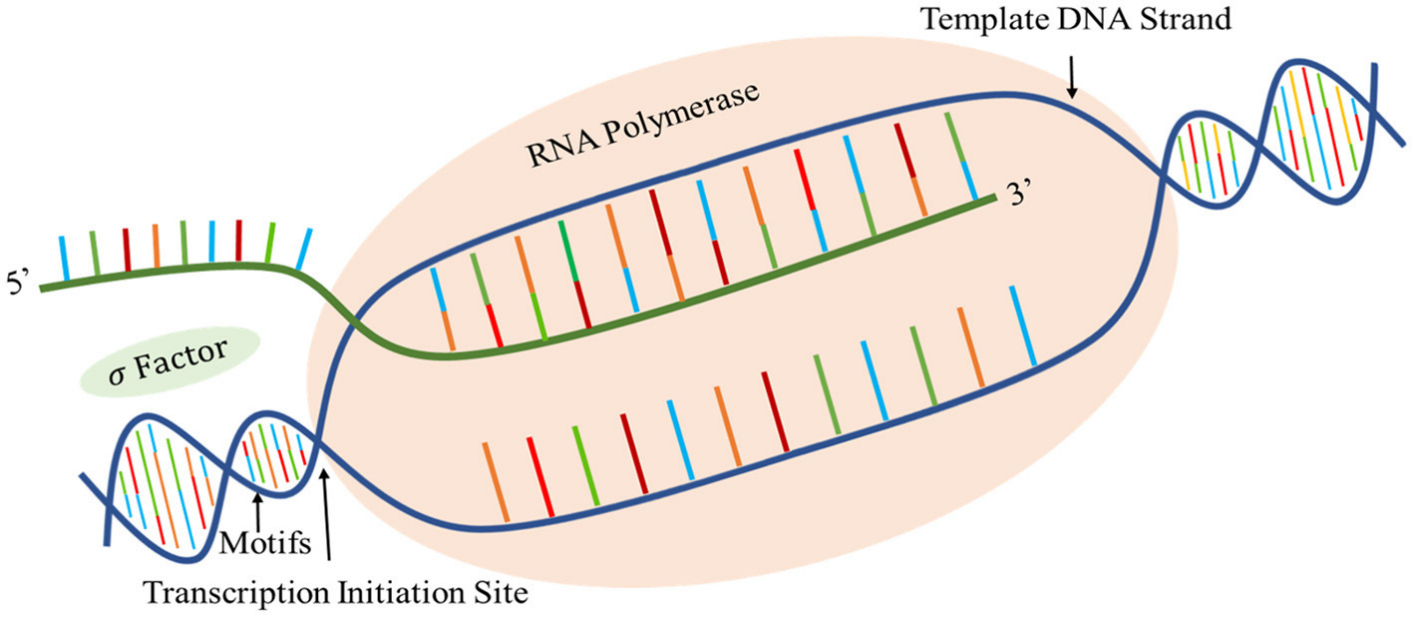
Schematic diagram of the prokaryotic promotor structure and its biological processes.

Agrobacterium belongs to the family of ubiquitous gram-negative soil bacteria. Infectious strains of agrobacterium such as agrobacterium tumefaciens strain C58 cause hairy root and crown gall diseases in plants (Goodner et al., 2001). Promotors are the genomic regions upstream of a gene on DNA where transcription factor and RNA polymerase bind together to initiate gene transcription (Sawadogo and Roeder, 1985; Zhao et al., 2017; Zhang et al., 2018). The biological process of prokaryotic promotors is shown in Figure 1. The study of promoters is the first step to understanding gene expression.

**Figure 2:**
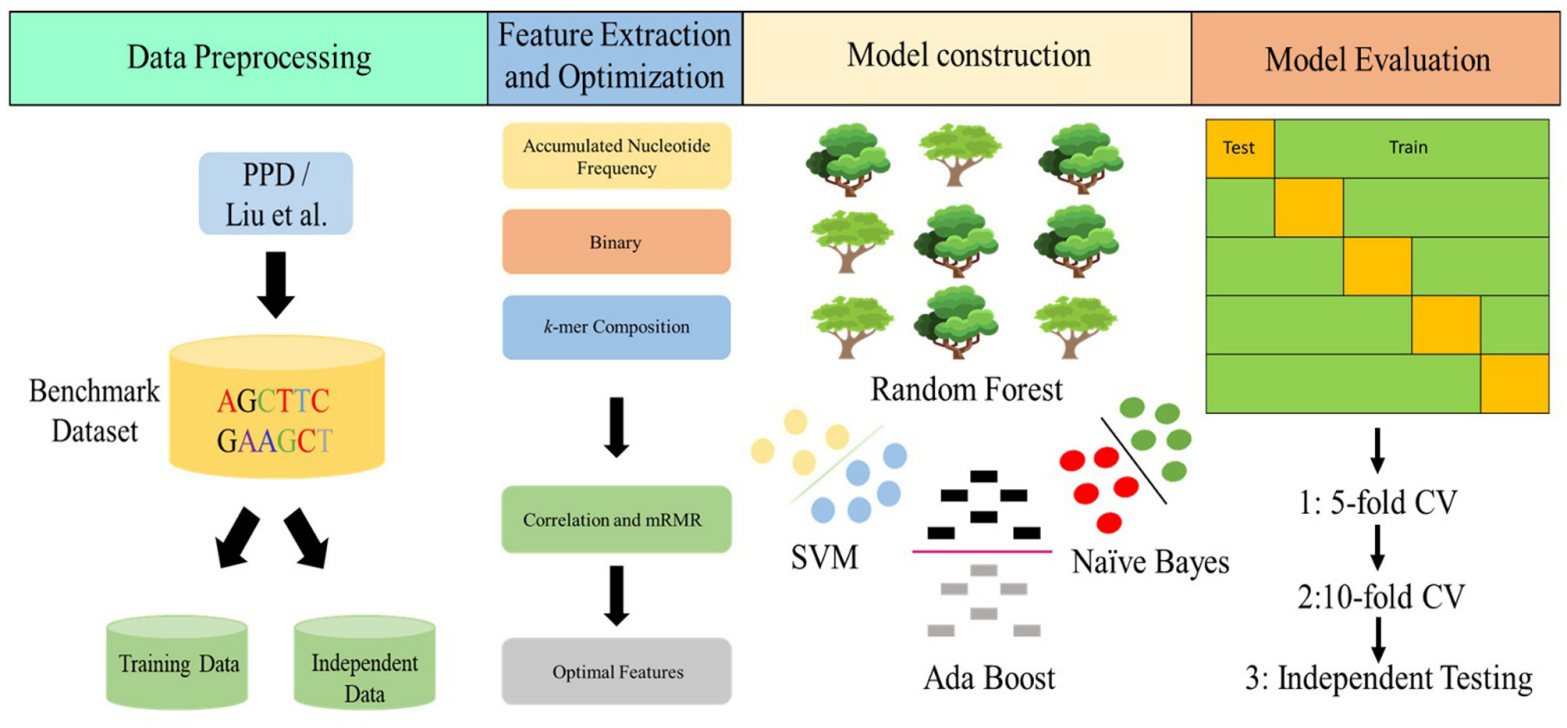
Overall workflow of the study.

Correct identification of the promotor sequence could produce vital signs for understanding its mechanism of the regulation (Cao et al., 2022; Li et al., 2022b). Currently, numerous tentative techniques, such as mass spectrometry (Flusberg et al., 2010), reduced-representation bisulfite sequencing (Doherty and Couldrey, 2014), and single-molecule real-time sequencing (Boch and Bonas, 2010), have been developed. Though these procedures are quite helpful in the identification of promotors prediction, they are costly when applied to large sequencing data. Thus, a bioinformatics tool to recognize the promotor sequence is urgently needed. At present, some computational tools have been presented to recognize promotors in multiple species, such as PePPer (de Jong et al., 2012) for *Escherichia coli* (*E.coli*) and *Bacillus subtilis* (*B.subtilis*); Promotech for *Bacillus amyloliquefaciens* (*B. amyloliquefaciens*) XH_7_ bacterium (Chevez-Guardado and Peña-Castillo, 2021); DeePromotors (Oubounyt et al., 2019) for TATA promotors (Zou et al., 2016) in eukaryotic genomes; iProEP (Lai et al., 2019) for *Homo sapiens* (*H. sapiens*), *Drosophila melanogaster* (*D. melanogaster*), *Caenorhabditis elegans* (*C. elegans*), *B. subtilis*, and *E. coli*; and iPromotor-2L (Liu et al., 2018) for bacterial promotors. However, there is no such model for *A. tumefaciens* C58 strain. To address the above-mentioned problems, we designed an RF-based model to predict promotor sequences in agrobacterium tumefaciens strain C58. Figure 2 illustrates the workflow of the projected model.

Accumulated nucleotide frequency, binary encodings, and *k*-mer nucleotide composition were utilized to convert sequences into numerical features, and then these features were optimized by using correlation and the mRMR-based feature selection algorithm. After this, these optimized features were inputted into a random forest classifier for the identification of promotor sequences on the basis of 10-fold cross-validation. As a result, an ideal model was attained.

## 2. Materials and methods

A precise and accurate dataset is necessary to establish a prediction model (Liang et al., 2017; Ning et al., 2021a,b; Su et al., 2021). Therefore, we obtained the experimentally verified *Agrobacterium tumefaciens* strain C58 promotors data of 706 sequences from PPD (http://lin-group.cn/database/ppd/index.php) and also collected negative data of 2860 sequences of 81 bp from (http://bioinformatics.hitsz.edu.cn/iPromotor-2L/data). Moreover, we divided the dataset into 80/20 ratios for training and testing the model.

### 2.1. Feature descriptors

Selecting the feature encodings that are useful and autonomous is a key stage in establishing machine learning-based models (Lv et al., 2021; Zhang D. et al., 2021; Ao et al., 2022a; Li et al., 2022a; Ning et al., 2022; Teng et al., 2022; Wei et al., 2022). Representing the DNA sequences with a mathematical manifestation is very important in functional element identification. Some DNA sequences coding strategies such as accumulated nucleotide frequency, physiochemical properties, binary encodings, nucleotide chemical properties and *k*-tuple nucleotide frequency component, nucleotide pair spectrum encoding, and natural vector have been applied in bioinformatics (Dao et al., 2020; Yang X. et al., 2021; Zhang Y. et al., 2021; Ao et al., 2022b; Ren et al., 2022). The performance of these feature descriptors was good. Here, to extract DNA sequence information as more as possible, accumulated nucleotide frequency, *k*-mer nucleotide composition, and binary encodings were presented to describe the DNA sequences based on their superior performance.

#### 2.1.1. Accumulated nucleotide frequency

The encoding of ANF consists of the distribution and frequency of nucleotides *n*_*i*_ in the sequences. The nucleotide density *D*_*i*_ at any position in the sequence can be calculated as follows:


(1) $$\begin{array}{l} D_{i}=\;\frac{1}{\left|n_{i}\right|}\;\sum_{k=1}^{z}f\left(n_{i}\right),\;f\left(g\right)=\;\{\begin{array}{c} 1\;if\;n_{i}=g\\ 0\;in\;other\;case \end{array}\; \end{array}$$

where *z* is the sequence length, *n*_*i*_ is the length of the string {*n*_1_, *n*_2_, …, *n*_*i*_} (Li et al., 2022c,d) in the sequence, and *g* ∈ {A, G, C, T}.

#### 2.1.2. k-mer nucleotide composition

*k-mer* nucleotide composition can reflect short-range nucleotide interaction of sequences (Salimi and Moeini, 2021; Zhang et al., 2022b; Dao et al., 2023). The nucleotide residues can be obtained *via* a sliding window method by setting the window size of *k* bp with a step size of 1 bp to examine a sequence with *n* bp. An arbitrary sample *Z* with the sequence length of *n* (where *n* is 81bp) can be characterized as


(2) $$\begin{array}{l} Z=\;Q_{1}\;Q_{2}\;Q_{3}\ldots ..\;Q_{i}\ldots ..\;Q_{\left(n-1\right)}\;Q_{n} \end{array}$$

where *Q*_*i*_ signifies the nucleotide {A, G, C, T} at the *i-*th position. The sequences can be transformed into the 4^*k*^ D vector using *k-mer* nucleotide composition as follows:


(3) $$\begin{array}{l} Q_{k}={\left[p_{1}^{k-tuple}p_{2}^{k-tuple}.\ldots .p_{i}^{k-tuple}\ldots ..p_{4^{k}}^{k-tuple}\right]}^{t} \end{array}$$

where *t* denotes the transposition of the vector, and $p_{1}^{k-tuple}$ symbolizes the occurrence of the *i-*th *k-mer* nucleotide composition in the sequence. When *k* = 1, a DNA sample can be decoded into a 4 D vector *Q*_1_ = [*p(A), p(T), p(G), p(C)*]^*t*^. When *k* = 2, the DNA sample can be described by a 16-dimension vector. In this study, the value of *k* was set as 4 due to the best results. The whole results of *k-*mer nucleotide composition (*k* = 1,2,3,4,5,6) on training and independent data are shown in Supplementary Table S1.

#### 2.1.3. Binary encoding

Encoding “0” and “1” can represent any information in the computational work (Zou et al., 2019). Therefore, we can directly convert a DNA sequence into a string of characters, which is consisted of “0” and “1.” A = (1,0,0,0), T = (0,1,0,0), G = (0,0,1,0), and C = (0,0,0,1). Thus, a DNA sample of 81 bp length is converted into a 324 (4 × 81) dimension vector in this study.

### 2.2. Feature selection

#### 2.2.1. Correlation

Feature selection is an important step for improving model performance (Dao et al., 2020). Correlation is a familiar comparison measure between two features. If two features are linearly dependent, then their correlation coefficient will be “±1.” If the features are uncorrelated, the correlation coefficient will be “0.” There are two comprehensive classes that can be used to measure the correlation between two random variables. One is based on information theory, and the other is classical linear correlation. The most familiar measure is the linear correlation coefficient. The linear correlation coefficient “*d*” for a pair of (*m, n*) variables is specified as


(4) $$\begin{array}{l} d=\frac{\sum \left(m_{i}-\;{\bar{m}}_{i})(n_{i}-\;{\bar{n}}_{i}\right)}{\sqrt{{\sum \left(m_{i}-\;{\bar{m}}_{i}\right)}^{2}}\;\sqrt{{\sum \left(n_{i}-\;{\bar{n}}_{i}\right)}^{2}}}\; \end{array}$$

Due to the expansion of the data, the correlation coefficient which is good for a sample may not produce decent outcomes for the whole population. Therefore, it is necessary to determine the significant association between the features, while captivating the whole population. The most commonly used method to examine statistical correlation is the *t*-test. The procedure used in the projected algorithm is to use the *t*-test for choosing the most important features from the whole feature set. The formula for calculating the suitable “*T*” value to test the consequence of a correlation coefficient employs the “*T*” distribution. The “*T*” value can be calculated as


(5) $$\begin{array}{l} T\;=\;d\;\sqrt{\frac{i-2}{1-\;d^{2}}}\; \end{array}$$

where “*i*” is the number of instances and “*d*” is the correlation coefficient for sample data. The significance of the relationship is expressed in probability levels: *p* (e.g., significant at *p* = 0.05). The degrees of freedom for entering the *T*-distribution are *i –* 2. If the value of “*T*” is higher than the threshold value at the 0.05 significant level, then the feature will be significant and selected (Zulfiqar et al., 2022a).

#### 2.2.2. mRMR

mRMR is a very popular feature selection technique, and it has been applied in many bioinformatics and biological applications (He et al., 2020; Zulfiqar et al., 2021b; Su et al., 2023). The compactness functions are described as “*i*” and “*y*,” and their corresponding probabilities are *P*(*i*) and *P*(*y*). The common information between these two functions can be defined as


(6) $$\begin{array}{l} Q_{\min}(f_{i},f_{y})=\sum_{i\in Q}\sum_{y\in Y}P\left(fi,\;fy\right)\log\frac{P(i,y)}{P\left(i\right),P(y)}) \end{array}$$

If the target is *J*_*i*_, then calculating the mutual information in relation to the target and can be defined as


(7) $$\begin{array}{l} Q_{\max}(f_{i},J_{i})=\sum_{fi\in Q}\sum_{Ji\in i}P\left(fi,\;Ji\right)\log\frac{P(fi,Ji)}{P\left(fi\right),P(Ji)}) \end{array}$$

Thus, *mRMR*(*f*_*i*_)can be calculated as


(8) $$\begin{array}{l} \text{mRMR}\left(f_{i}\right)=\frac{Q_{max}\left(f_{i},J_{i}\right)}{Q_{min}\left(f_{i},f_{y}\right)} \end{array}$$

### 2.3. Machine learning classifiers

Naïve Bayes (NB) classifier has been used widely in bioinformatics due to its simplicity (Ye et al., 2021). This classification method totally depends on the Bayes theorems. Ada boost (AB) is another popular machine learning technique. The main idea of AB is to set the classifiers' weights and trained the data in each and every iteration. The support vector machine (SVM) is also very famous and has been used in many bioinformatics and computational biology-related tools (Tao et al., 2020; Ahmed et al., 2022; Manavalan and Patra, 2022; Zou et al., 2022; Bupi et al., 2023; Zulfiqar et al., 2023). It is mostly used to perform binary classification. We implemented these algorithms in Weka version 3. 8.4. by using the default values. RF is a combined knowledge algorithm and is widely used in bioinformatics (Ao et al., 2022c; Zhang et al., 2023). The main idea of this is to combine several weak classifiers and outcomes generated on the basis of voting. The brief description is clearly described by Zulfiqar et al. (2021a). We have used randomized and grid search cross-validations to tune the hyperparameters. We executed this job in the Scikit-learn package version 0.22.2, and its parameters are summarized in Table 1. All experiments were carried out on a Windows operating system with 1.7 GHz intel quad-core i5.

**Table 1:** Best parameters of the proposed model.

**Best parameters**	
“N-estimators”	80
“Max_depth”	20
“Bootstrap”	True
“Min_samples_leaf”	1
“Min_samples_split”	2

**Algorithm 1:** Correlation and mRMR-based Feature Selection Algorithm.

**Input:** Training data: = H (*x_1_, x_2_*, ……, *x_*k*_, x_*c*_*)
**Output:** H_best_
**1**^**st**^ **Round**
1 Start
2 **for** *i* =1 to *k* **do**
3 *d* = calculate correlational coefficient (*x_*i*_, x_*c*_*)
**end**
4 let *p* = 0.05 significant level
5 let ρ = 0 / suppose there is no significant correlation between *f_*i*_* and *f_*c*_*
6 **for** *i* = 1 to *k* **do**
*q* = calculate the significance (*d, ρ*) for *x_*i*_* / by using the *T-test*
7 **if** *T* > CV / critical value
8 H_best_ = H_list_
9 **end**
10 return H_best_
**2**^**nd**^ **Round**
11 Start
12 By sorting the features
13 **for** each feature *fi* in *Z* **do**
14 By calculating the mutual information in relation to other features as
15 $Q_{\min}\left(f_{i},f_{y}\right)=\underset{i\in Q}{\sum }\underset{y\in Y}{\sum }P\left(fi,\;fy\right)\log\frac{P\left(i,y\right)}{P\left(i\right),P\left(y\right)}$)
16 By calculating the mutual information in relation to the target:
17 $Q_{\max}\left(f_{i},J_{i}\right)=\underset{fi\in Q}{\sum }\underset{Ji\in i}{\sum }P\left(fi,\;Ji\right)\log\frac{P\left(fi,Ji\right)}{P\left(fi\right),P\left(Ji\right)}$)
18 By calculating the *mRMR(f*_*i*_*)* as
19 *mRMR(f*_*i*_*)* = $\frac{Q_{\max}\left(f_{i},J_{i}\right)}{Q_{\min}\left(f_{i},f_{y}\right)}$
20 **end**
21 **for** by sorting the features in descending order
22 By updating the matrix *Z'* with sorted features
23 **end**
24 return *Z'*

### 2.4. Evaluation metrics

Accuracy, precision, recall, and F1 (Hasan et al., 2020; Zhang et al., 2020; Wei et al., 2021b; Shoombuatong et al., 2022; Yang et al., 2022; Zulfiqar et al., 2022b) were employed to assess the performance of the prediction model and are expressed as


(9) $$\begin{array}{l} \{\begin{array}{l} Acc=\frac{tp\;+\;tn}{tp+fp\;+\;tn+fn}\\ Pre=\frac{tp}{tp\;+fp}\;\\ Rec=\frac{tp}{tp\;+fn}\;\\ F1=2\times \;\frac{Pre\;\times \;Rec}{Pre\;+\;Rec} \end{array} \end{array}$$

where *tp* symbolizes the correctly predicted promotor sequences and *fp* signifies the non-promotor sequences classified as the promotor sequence. On the other hand, *tn* represents the correctly identified non-promotor sequences, and *fn* demonstrates the promotor sequences, which were classified as the non-promotor sequence.

## 3. Results and discussion

### 3.1. Performance evaluation

**Figure 3:**
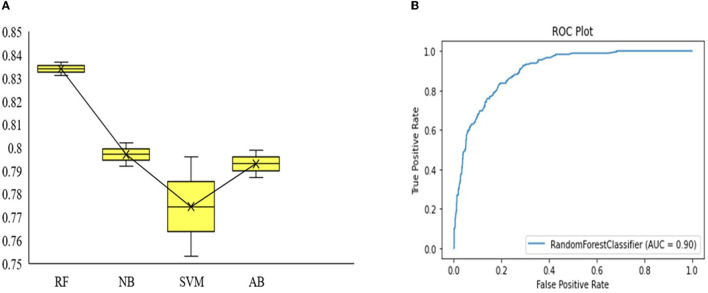
Performance comparison on different machine learning classifiers by using training and independent datasets. The higher point represents the training accuracy and the lower point represents the accuracy on independent data **(A)**. *AUC* curve of the anticipated model **(B)**.

On the basis of sequence features, we constructed an anticipated model to recognize promotor sequences in *A. tumefaciens* C58 strain. First, the training data were converted into numerical feature vectors using accumulated nucleotide frequency, binary encodings, and *k*-mer nucleotide composition. After this, these features were optimized by using correlation and the mRMR-based algorithm. First, correlation measures and then mRMR were used to select the finest feature subset for the improved prediction outcomes. Afterward, these features were inputted into four machine learning methods. Cross-validation (CV) is a statistical analysis procedure and has been applied in machine learning to evaluate the model's performance (Yang H. et al., 2021; Chen et al., 2022; Liao et al., 2022; Xiao et al., 2022; Zhang et al., 2022a; Yang et al., 2023). In this study, the 10-fold CV test was used to investigate the performance of machine learning methods. In 10-fold CV, the benchmark dataset was randomly separated into ten groups of about equal size. Each group was individually tested by the model which trained with the remaining nine groups. Therefore, the 10-fold CV method was performed 10 times, and the average of the results was the final result (Charoenkwan et al., 2021; Wei et al., 2021a; Hasan et al., 2022). We have trained 32 models on AB, SVM, NB, and RF. At first, we used single encodings and their fusion to train and test the models, and then we optimized the feature encodings and their fusions by using correlation and the mRMR-based algorithm. In this phase, we utilized the *t-*test and picked the significant features by selecting the probability of the significance relation 0.05, and then used mRMR and picked the top features. Moreover, we inputted these features into AB, SVM, NB, and RF and found that the performance of *k*-mer was good as compared to other feature encodings and their fusion. The accuracy of *k*-mer in RF was 3.5%−4.1% higher than the other three classifiers. The *AUC* curve of the anticipated model was 0.900. The *accuracy, precision, recall, and F1* are recorded in Table 2. The performance comparison on different machine learning classifiers by using training and independent datasets and *ROC* plot of the anticipated model is shown in Figures 3A, B.

**Table 2:** Performance of models using different classifiers on the training and independent dataset.

	**Training dataset**							**Independent dataset**					
**Classifier**	**FS**	***k***	**Method**	**Accuracy**	**Precision**	**Recall**	**F1**	**AUC**	**Accuracy**	**Precision**	**Recall**	**F1**	**AUC**
AB	256	4	*k*-mer	0.761	0.772	0.761	0.791	0.812	0.775	0.820	0.801	0.798	0.881
	50	4	*k*-mer	0.799	0.802	0.785	0.789	0.856	0.787	0.824	0.799	0.805	0.872
	324		Binary	0.738	0.742	0.756	0.712	0.786	0.700	0.702	0.700	0.730	0.765
	48		Binary	0.745	0.742	0.698	0.789	0.820	0.720	0.732	0.702	0.726	0.789
	82		ANF	0.684	0.645	0.689	0.743	0.731	0.641	0.692	0.688	0.655	0.699
	38		ANF	0.743	0.726	0.775	0.746	0.796	0.696	0.702	0.698	0.710	0.756
	662		Fusion	0.745	0.732	0.785	0.775	0.799	0.720	0.732	0.775	0.745	0.774
	136		Fusion	0.778	0.768	0.792	0.800	0.845	0.738	0.745	0.765	0.725	0.806
SVM	256	4	*k*-mer	0.761	0.802	0.789	0.799	0.865	0.749	0.838	0.761	0.648	0.860
	50	4	*k*-mer	0.796	0.802	0.802	0.812	0.883	0.753	0.748	0.753	0.756	0.832
	324		Binary	0.744	0.747	0.778	0.765	0.792	0.725	0.755	0.760	0.763	0.786
	48		Binary	0.774	0.775	0.732	0.778	0.815	0.748	0.800	0.778	0.769	0.845
	82		ANF	0.666	0.697	0.732	0.705	0.766	0.612	0.623	0.633	0.605	0.699
	38		ANF	0.755	0.768	0.748	0.759	0.820	0.695	0.703	0.713	0.705	0.806
	662		Fusion	0.710	0.722	0.708	0.709	0.745	0.705	0.700	0.700	0.710	0.740
	136		Fusion	0.752	0.759	0.758	0.768	0.801	0.741	0.750	0.770	0.765	0.810
NB	256	4	*k*-mer	0.748	0.780	0.778	0.719	0.823	0.788	0.801	0.799	0.802	0.884
	50	4	*k*-mer	0.802	0.821	0.823	0.827	0.881	0.792	0.778	0.792	0.802	0.878
	324		Binary	0.737	0.775	0.765	0.789	0.794	0.776	0.770	0.778	0.793	0.835
	48		Binary	0.777	0.789	0.759	0.788	0.864	0.782	0.810	0.815	0.816	0.891
	82		ANF	0.675	0.689	0.720	0.696	0.756	0.665	0.685	0.691	0.701	0.741
	38		ANF	0.735	0.741	0.728	0.733	0.770	0.723	0.715	0.705	0.740	0.762
	662		Fusion	0.712	0.754	0.726	0.745	0.768	0.764	0.777	0.756	0.750	0.788
	136		Fusion	0.778	0.802	0.808	0.810	0.880	0.790	0.807	0.803	0.800	0.892
RF	256	4	*k-mer*	0.809	0.830	0.810	0.74	0.861	0.808	0.841	0.811	0.799	0.897
	50	4	*k*-mer	0.837	0.840	0.841	0.801	0.900	0.831	0.842	0.837	0.818	0.900
	324		Binary	0.792	0.632	0.792	0.701	0.842	0.784	0.804	0.808	0.788	0.887
	48		Binary	0.796	0.653	0.801	0.732	0.865	0.806	0.825	0.811	0.806	0.892
	82		ANF	0.791	0.630	0.791	0.702	0.850	0.788	0.803	0.773	0.778	0.878
	38		ANF	0.795	0.642	0.789	0.743	0.866	0.794	0.726	0.792	0.80	0.868
	662		Fusion	0.792	0.630	0.790	0.708	0.822	0.794	0.771	0.790	0.789	0.856
	136		Fusion	0.801	0.786	0.795	0.800	0.881	0.807	0.799	0.820	0.812	0.889

## 4. Conclusion

Promotors have a significant role in the transcription process because they are located on upstream of genes where RNA polymerase binds with the transcription factor and initiate the transcription. In this study, an RF model was established to identify promotors sequences in agrobacterium tumefaciens strain C58. In the proposed model, sequences were encoded using accumulated nucleotide frequency, *k*-mer nucleotide composition, and binary encodings and then optimized with correlation and the mRMR-based algorithm. After this, these optimized features were inputted into the RF-based classifier using the 10-fold CV test and achieved the best model. The estimated outcomes on independent data showed that the projected model provided brilliant performance and oversimplification. We provided the source codes and data freely at https://github.com/linDing-groups/model_promotor. Researchers can yield good results for DNA sequences and recognize their roles by using our freely available source codes. In future, we will further improve the efficiency by using CNN/GNN and release a webserver to make our anticipated model more convenient for users without mathematical and programming knowledge.

## Data availability statement

The original contributions presented in the study are included in the article/Supplementary material, further inquiries can be directed to the corresponding authors.

## Author contributions

HZ: conceptualization, supervision, methodology, experimentation, visualization, and writing—original draft preparation. ZA and BK: data curation and methodology. FH: data curation. Z-YZ: supervision, methodology, reviewing, and editing. FL: supervision, reviewing, and editing. All authors have read and agreed to the published version of the manuscript.
