# Supplementary material for: Computational prediction of promotors in Agrobacterium tumefaciens strain C58 by using the machine learning technique
Source: Front Microbiol. 2023 Apr 13;14:1170785. doi: 10.3389/fmicb.2023.1170785 (PMC10133480; doi:10.3389/fmicb.2023.1170785)
Supplement: Supplementary file 1 [file Table_1.DOCX]

**Table 1S.** Performance of *k*-mer nucleotide composition using different classifiers on Training and Independent datasets.

|  | *Training Data Independent Data* | | | | | | | | |  |  |
| --- | --- | --- | --- | --- | --- | --- | --- | --- | --- | --- | --- |
| Classifier |  | *k* |  | *Accuracy* | *Precision* | *Recall* | *F1* | *Accuracy* | *Precision* | *Recall* | *F1* |
| AB |  | 1 |  | 0.756 | 0.772 | 0.767 | 0.773 | 0.765 | 0.759 | 0.781 | 0.788 |
|  |  | 2 |  | 0.729 | 0.782 | 0.775 | 0.772 | 0.767 | 0.754 | 0.771 | 0.765 |
|  |  | 3 |  | 0.638 | 0.642 | 0.656 | 0.652 | 0.710 | 0.702 | 0.700 | 0.730 |
|  |  | 4 |  | 0.799 | 0.802 | 0.785 | 0.789 | 0.787 | 0.804 | 0.799 | 0.805 |
|  |  | 5 |  | 0.674 | 0.625 | 0.639 | 0.643 | 0.638 | 0.690 | 0.698 | 0.675 |
|  |  | 6 |  | 0.741 | 0.736 | 0.763 | 0.740 | 0.716 | 0.732 | 0.748 | 0.740 |
| SVM |  | 1 |  | 0.764 | 0.782 | 0.784 | 0.789 | 0.751 | 0.763 | 0.757 | 0.777 |
|  |  | 2 |  | 0.786 | 0.803 | 0.806 | 0.809 | 0.766 | 0.758 | 0.751 | 0.761 |
|  |  | 3 |  | 0.747 | 0.744 | 0.776 | 0.768 | 0.731 | 0.757 | 0.763 | 0.771 |
|  |  | 4 |  | 0.796 | 0.802 | 0.802 | 0.812 | 0.753 | 0.778 | 0.783 | 0.796 |
|  |  | 5 |  | 0.667 | 0.681 | 0.693 | 0.705 | 0.662 | 0.648 | 0.652 | 0.648 |
|  |  | 6 |  | 0.751 | 0.758 | 0.758 | 0.769 | 0.709 | 0.703 | 0.717 | 0.715 |
| NB |  | 1 |  | 0.768 | 0.781 | 0.788 | 0.759 | 0.758 | 0.761 | 0.769 | 0.792 |
|  |  | 2 |  | 0.792 | 0.820 | 0.803 | 0.817 | 0.791 | 0.783 | 0.791 | 0.800 |
|  |  | 3 |  | 0.738 | 0.747 | 0.755 | 0.749 | 0.726 | 0.740 | 0.738 | 0.723 |
|  |  | 4 |  | 0.802 | 0.821 | 0.823 | 0.827 | 0.792 | 0.798 | 0.792 | 0.802 |
|  |  | 5 |  | 0.695 | 0.699 | 0.727 | 0.700 | 0.662 | 0.675 | 0.689 | 0.691 |
|  |  | 6 |  | 0.744 | 0.743 | 0.738 | 0.753 | 0.728 | 0.722 | 0.712 | 0.751 |
| RF |  | 1 |  | 0.811 | 0.821 | 0.810 | 0.794 | 0.803 | 0.826 | 0.801 | 0.801 |
|  |  | 2 |  | 0.817 | 0.835 | 0.838 | 0.804 | 0.808 | 0.829 | 0.831 | 0.808 |
|  |  | 3 |  | 0.799 | 0.773 | 0.762 | 0.797 | 0.780 | 0.784 | 0.805 | 0.803 |
|  |  | 4 |  | 0.837 | 0.840 | 0.841 | 0.821 | 0.831 | 0.842 | 0.837 | 0.818 |
|  |  | 5 |  | 0.784 | 0.773 | 0.792 | 0.762 | 0.773 | 0.800 | 0.781 | 0.780 |
|  |  | 6 |  | 0.798 | 0.798 | 0.809 | 0.797 | 0.792 | 0.806 | 0.798 | 0.796 |
